# Work, Fresh Air, and Exercise
†Work, Fresh Air, and Exercise: Essential to Health and Happiness. A lecture delivered at the request of the Torquay Association for early closing. By C. Radclyffe Hall, M.D. Pamphlet. Torquay: 1857.


**Published:** 1857-10

**Authors:** 


### WORK, FRESH AIR, AND EXERCISE.†

The Torquay Early Closing Association have an earnest and able associate in Dr. Radclyffe Hall. He gives in this lecture

† Work, Fresh Air, and Exercise: Essential to Health and Happiness. A lecture delivered at the request of the Torquay Association for early closing. By C. Radclyffe Hall, M.D. Pamphlet. Torquay: 1857.

a very complete popular outline of the human physiology, so as to show the necessity of fresh air and exercise for maintaining health and happiness. And, therefore, because these are indissolubly joined, he enforces on the minds of his business hearers and readers to close their establishments at seven o'clock, so that "two or three hours might be given for them and their assistants, male or female, to take out-door exercise; to hold cheerful converse; to ventilate their thoughts as well as their bodies." Dr. Hall delivered this lecture to the inhabitants of one of the most favoured spots in the island for pure air: but even more forcibly must his remarks apply to the denizens of our crowded inland towns and cities. Such would do well to bear in mind, and act up to the following aphorisms:

"Nothing can adequately supplant out-door exercise, especially at that age when nature is finishing her masterpiece of creation—man —in his growth and development. If we want a future race of men to maintain old England's greatness—stint not the rising generation in fresh air. If you want healthy fathers—fresh air. If healthy mothers—fresh air. If you want good men at their work—fresh air. If you want good humoured and hearty wives—fresh air. If you wish your sons to be good looking, and your daughters handsome—give them fresh air and exercise. If you wish for peevishness, irritability, weakness and want, suffering and sorrow—neglect this truth. Be less happy than your Creator made you to be. Sin against knowledge, and regret it too late."

